# Single nuclei profiling identifies cell specific markers of skeletal muscle aging, frailty, and senescence

**DOI:** 10.18632/aging.204435

**Published:** 2022-12-13

**Authors:** Kevin Perez, Serban Ciotlos, Julia McGirr, Chandani Limbad, Ryosuke Doi, Joshua P. Nederveen, Mats I. Nilsson, Daniel A. Winer, William Evans, Mark Tarnopolsky, Judith Campisi, Simon Melov

**Affiliations:** 1 Buck Institute for Research on Aging, Novato, CA 94952, USA; 2 Drug Discovery Research, Astellas Pharma, Tsukuba, Ibaraki, Japan; 3 Department of Pediatrics, McMaster University, Ontario, Canada; 4 Exerkine Corporation, Hamilton, Canada; 5 Department of Nutritional Sciences and Toxicology, University of California, Berkeley, CA 94720, USA

**Keywords:** aging, transcriptomics, muscle, senescence, sarcopenia

## Abstract

Aging is accompanied by a loss of muscle mass and function, termed sarcopenia, which causes numerous morbidities and economic burdens in human populations. Mechanisms implicated in age-related sarcopenia or frailty include inflammation, muscle stem cell depletion, mitochondrial dysfunction, and loss of motor neurons, but whether there are key drivers of sarcopenia are not yet known. To gain deeper insights into age-related muscle loss, we performed transcriptome profiling on lower limb muscle biopsies from 72 young, elderly, and frail human subjects using bulk RNA-seq (*N* = 72) and single-nuclei RNA-seq (*N* = 17). This combined approach revealed changes in gene expression that occur with age and frailty in multiple cell types comprising mature skeletal muscle. Notably, we found increased expression of the genes *MYH8* and *PDK4*, and decreased expression of the gene *IGFN1*, in aged muscle. We validated several key genes changes in fixed human muscle tissue using digital spatial profiling. We also identified a small population of nuclei that express *CDKN1A*, present only in aged samples, consistent with p21^cip1^-driven senescence in this subpopulation. Overall, our findings identify unique cellular subpopulations in aged and sarcopenic skeletal muscle, which will facilitate the development of new therapeutic strategies to combat age-related frailty.

## INTRODUCTION

Age is the largest risk factor for developing sarcopenia - a loss of skeletal muscle mass and function [1]. Individuals over the age of 50 typically lose ~1% of muscle mass per year [2, 3]. Currently, physical activity appears to be the only therapy for sarcopenia, but its effectiveness declines with advanced age [4, 5], and is only moderately successful. Loss of muscle mass can further cause frailty, and increase the likelihood of falls and fractures [6]. The major mechanisms that drive age-related loss of muscle mass and function are unclear - although inflammation, impaired muscle regeneration due to loss of stem cells (satellite cells), loss of motor neurons and mitochondrial dysfunction have all been implicated [7]. Throughout this manuscript, we use the term “sarcopenia” and “frail” interchangeably, implying individuals who are demonstrably functionally impaired relative to age-matched controls.

To better understand potential mechanisms driving sarcopenia and frailty in old age, several studies have examined changes in skeletal muscle gene expression with age. Differential expression with age has been reported for genes encoding proteins that participate in mitochondrial function, muscle structure and inflammation (e.g., mitochondrial ribosomal proteins, myosin heavy chain and IL-6, respectively). Many such studies used bulk RNA sequencing and modest sample sizes [8, 9]. Changes in muscle fiber types, notably a reduction in type 2 fibers, is also a characteristic of aged skeletal muscle [10]. Several aspects of the type 2 fiber gene expression profile can be reversed by exercise which confers functional improvement [11, 12].

At the cellular level, senescence is a cell fate that typically entails cell cycle arrest and a complex senescence-associated secretory phenotype (SASP) that can be induced by myriad stresses, including activated oncogenes, DNA damage, reactive oxygen species (ROS) and certain genotoxic chemotherapeutics [13]. Senescent cells increase with age in many tissues [14], and have been hypothesized to contribute to sarcopenia [15]. In addition, the SASP includes many pro-inflammatory factors that can cause chronic inflammation and alter tissue microenvironments to fuel the development of age-related diseases [16]. Several markers of senescent cells have been identified in various tissues and include the cyclin-dependent kinase (CDK) inhibitors p16^INK4a^ (*CDKN2A*) and p21^cip1^ (*CDKN1A*), which orchestrate proliferative arrest. However, there are no universally agreed upon drivers or biomarkers of senescence in any tissue, and senescent cells are generally present at very low numbers (<1–5%) [17]. A large-scale effort is underway to map senescent cells and define markers for multiple human and mouse tissues with age (https://sennetconsortium.org).

Single-cell analyses are improving our understanding of pathophysiology, including age-related diseases, by deconvolving the heterogeneity in cellular composition at the tissue level [18]. Muscle “single cell” analyses have been particularly challenging to perform due to the nature of the myofiber: a syncytium containing thousands of myonuclei. Several pioneering studies using single cell technologies have been recently performed, to gain better insight into biology of stemness, development, and aging [19–25]. The tissue organization of muscle practically prevents the preparation of “single cells” from muscle using conventional single cell workflows. Alternatively, nuclei from frozen tissue can be used to generate transcriptomic data [26–28]. This approach enables expression profiling of nuclei from muscle, despite its syncytial structure. Myonuclei within any muscle fiber express genes specific for different work capacities. Further, myofibers are broadly classified into two main types: type I (slow) and type II (fast) fibers. In addition to specific fiber types, skeletal muscle contains numerous other support cells, including endothelial cells, satellite cells, fibro-adipogenic precursor cells and infiltrating immune cells [25, 29, 30]. Bulk RNA-seq approaches can only provide a combined and aggregated view of the gene expression changes across all fiber and cell types.

Here, we contrast bulk RNA profiling of 72 biopsies from the lower limb muscles of young, old, and older frail human subjects, together with single nuclei sequencing (snuc-Seq) of 17 independent young and old muscle samples. Using the new technology of digital spatial profiling, which reports gene expression values at the genome wide level within the context of tissue microarchitecture [31], we also validate and localize several genes within individual fibers of young versus older subjects. This combined approach provides a more complete insight into the heterogeneity of both cell composition and gene expression in aged individuals of differing functional capacities, and is generally applicable to other tissues. The methodology is also particularly useful for enumerating changes in cell frequency with age, a fundamental outcome of aging. Finally, we identified unique populations of cells in aged versus frail muscle, including senescent cell types. Our results uncover potential new targets for therapies to treat age-related muscle loss and sarcopenia.

## RESULTS

### Cohort description and clinical characteristics

We isolated 20–50 mg biopsies from the *vastus lateralis* of 72 adult men [11, 32], including 19 young subjects (avg. age = 20 years) and 53 older subjects (avg. age = 75 years). The older subjects were subsequently classified into non-sarcopenic (*N* = 29) and sarcopenic (*N* = 24) subjects based on a variety of functional criteria. Given that there is at present no consensus on the definition of Sarcopenia, we used criteria that are most often used to assess populations thought to be sarcopenic [33] (see Supplementary Materials). These criteria included both functional and strength assessments. On average, older non-frail subjects had lower short physical performance battery (SPPB) test scores (*p* < .05), longer ‘Timed up and Go’ performances scores (*p* < .05), lower grip strengths (*p* < .05), Biodex determined isometric knee extension torque (*p* < .05) and leg press scores (*p* < .05) relative to younger subjects. Sarcopenic or frail older subjects had even more impaired SPPB scores (*p* < .05), longer ‘Timed up and Go’ performances (*p* < .05), lower grip strengths (*p* < .05), isometric knee extension torque (*p* < .05) and leg press scores (*p* < .05) relative to older non-frail subjects (Supplementary Figure 1, Supplementary Table 1).

### Changes in bulk gene expression with age and frailty in human muscle

**Figure 1:**
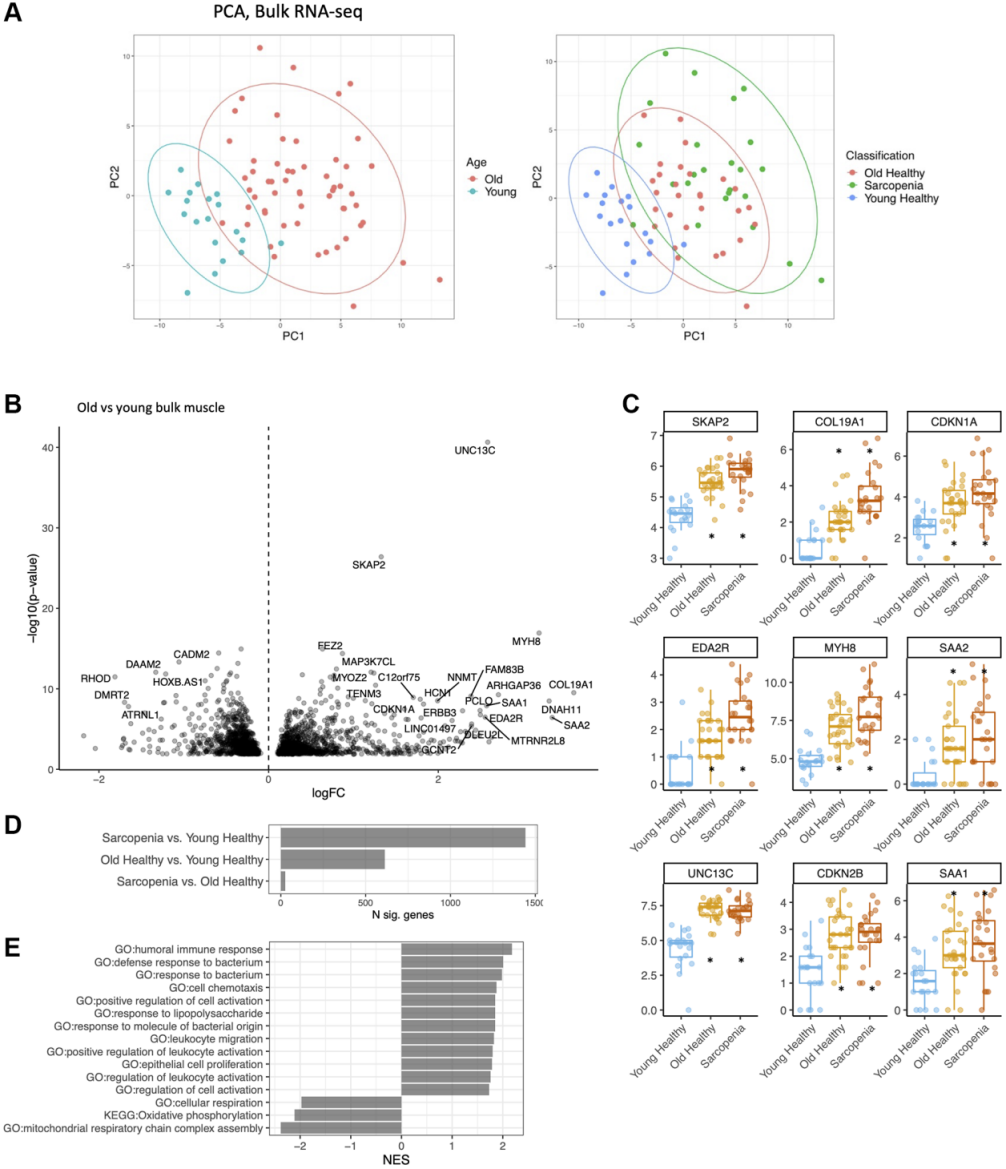
**Bulk RNA-seq identifies major gene expression changes in muscle with age.** (**A**) Principal component analysis (PCA) of bulk young, old and frail skeletal muscle. (left) Young (less than 20 years old) in blue, old (more than 65 years old) in red. (right) Young (blue), old (red), frail subjects (green). (**B**) Volcano plot of expression changes in old vs. young muscle. Labelled top 30 by abs (logFC) × -loglO (*p*-value). (**C**) Log (CPM) of MYH8, COL19Al, MTRNL8, CDKNlA, CDKN2B, AREG in young (green), old (blue) and frail subjects (red). Boxplot shows 25% percentile, 75% percentile and median. Stars were added when significant compared to young healthy (*q* < .01). (**D**) Number of DEGs per comparison. (**E**) Pathway analysis of dysregulated genes with age using KEGG, GO database (GSEA).

After quality control and processing using a standard RNA-seq pipeline (see Supplementary Materials), we performed bulk RNA sequencing on biopsies from all 72 subjects. We then performed principal component analysis (PCA) on the resultant gene expression datasets (Figure 1A). These analyses showed that gene expression at the bulk RNA level was largely distinct between younger versus older subjects (Figure 1A, Supplementary Table 1). We found 1442 differentially expressed genes (DEGs) between young versus sarcopenic, 613 between young versus old, and only 26 between old versus sarcopenic (Figure 1D). Samples from the older age group were marked by a statistically significant upregulation in the expression of several genes, including *MYH8*, *COL19A1, EDA2R, CDKN1A* and *CDKN2B* (Figure 1B, 1C). Conversely, expression of the following genes was lower with age: *IGFN1, MTND3P10, ATRNL1* and *PVALB*. Pathway analysis of the bulk data revealed several upregulated pathways of immune response, and bacterial response and downregulation of mitochondrial respiration (Figure 1E). Sarcopenic and old muscle had similar signatures, compared to young muscle (Supplementary Figure 2A) (R^2^ = 0.9), but MYH8 was higher in the samples from sarcopenic compared to non-sarcopenic (*Q* < .05).

To determine whether the aging signature we observed in human skeletal muscle was conserved across mammalian species, we compared our results to a published multi-tissue rat aging gene expression study [34]. Genes commonly upregulated in aged human and rat muscle included *CDKN1A, EDA2R, MUSK* and *CDKN2B* (Supplementary Figure 2B). Comparing our muscle aging signatures with a published plasma proteome aging signature [35], we determined that *CXCL11, EDA2R, MUSK* and *CXCL9* were commonly upregulated with age (Supplementary Figure 2C). We also compared the genes that we found different between non-sarcopenic and sarcopenic subjects to a published signature of sarcopenia from subjects of Chinese descent in Singapore [36], but we did not observe any overlap (data not shown). These findings suggest frailty markers may differ depending on ethnicity and tissue type.

### Genes associated with functional performance and age

We next asked whether these changes in gene expression were associated with clinical measures of function. Because age highly influenced most of the clinical metrics, we restricted this analysis to older subjects. Maintenance of muscle strength is generally considered more relevant to overall health at older ages [37]. For each clinical metric, we classified the subjects as good performers (greater than the mean) or poor performers (lower than the mean). Leg press was the clinical metric associated with the most changes in gene expression, and grip strength was associated with the least changes. *RPL10P9* was upregulated in good performers of SPPB and ‘Timed up and Go’, whereas *PNPLA3* was downregulated in good performers of isometric knee extension torque and leg press (Supplementary Table 2). Several genes were associated with both aging and muscle function. Indeed, one of the top downregulated genes with age - *IGFN1* -- was also upregulated in good performers of ‘Timed up and Go’. Similarly, *COL19A1*, one of the top upregulated genes with age, was downregulated in those with strong knee extensors (high isometric knee extension torque) (Supplementary Table 2). These associations with both age and muscle function at older ages suggest a functional role for these genes in muscle decline, and therefore may serve as proxies for muscle function in the elderly.

### Single-nuclei sequencing reveals 7 clusters of unique cell types

To determine how cell composition changes with age in muscle, we analyzed 17 independent biopsies from the vastus lateralis of younger and older individuals (6 young, 11 old) using snuc-Seq. Single nuclei sequencing has several advantages in the context of tissues comprised of multiple cell types and syncitia. Many single cell sequencing experiments rely on complex enzymatic digestion procedures to isolate cells of interest from the tissue. Such procedures can alter gene expression. Thus, tissue snap frozen at the time of isolation may best preserve the “*in vivo*” status of gene or protein expression. Here, we also employed a dedicated instrument for rapid extraction of nuclei from snap frozen fresh muscle tissue (Singulator, S2 Genomics) to rapidly isolate nuclei from the biopsies, thereby minimizing potential changes in gene expression resulting from conventional nuclei extraction procedures [38]. We then carried out snuc-Seq using a 10× workflow to interrogate individual cell types in skeletal muscle.

**Figure 2:**
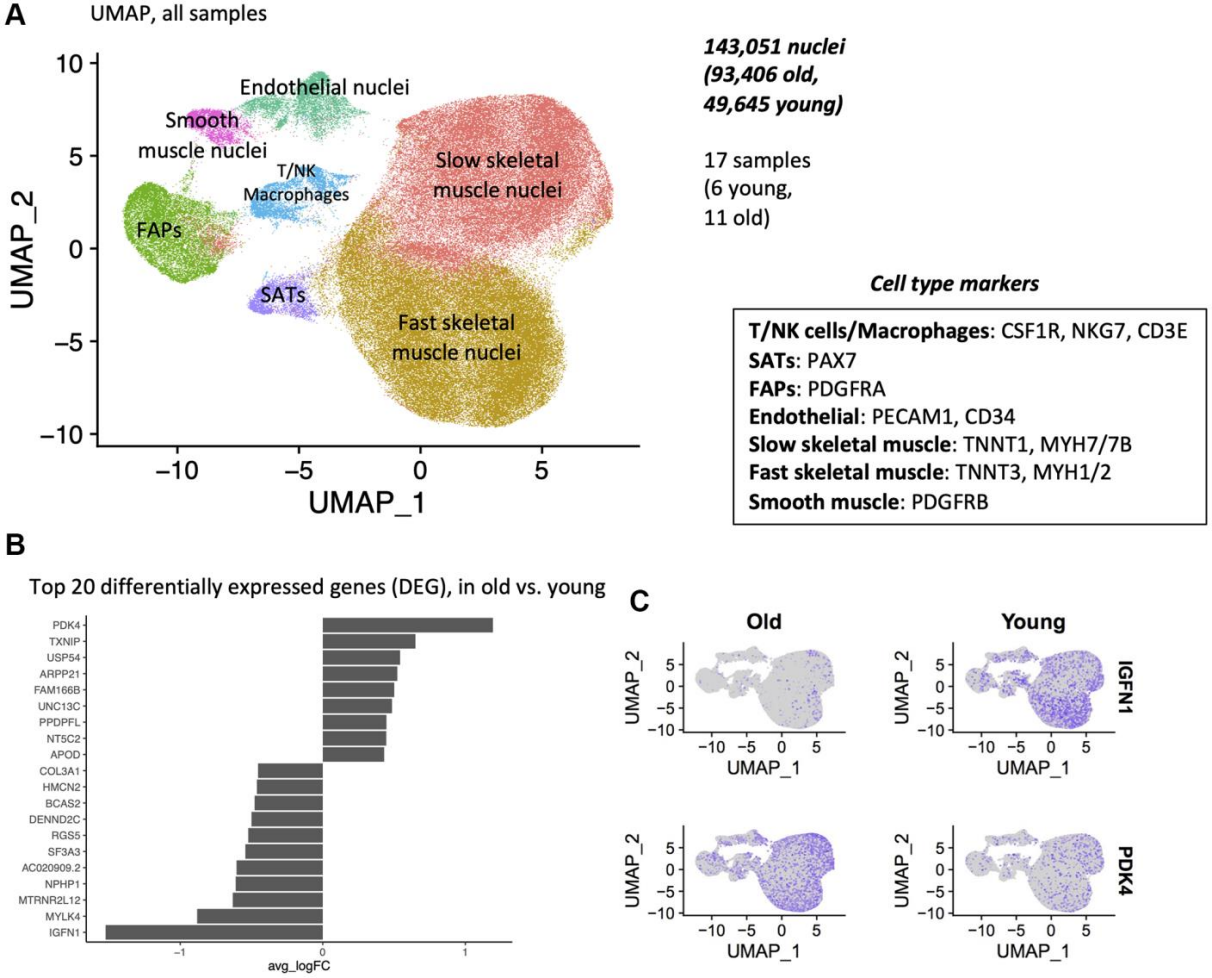
**Single-nuclei sequencing reveals 7 clusters of unique cell types, and differential gene expression with age.** (**A**) Uniform Manifold Approximation and Projection (UMAP) of 5′ single nuclei sequencing of human muscle. All samples are shown, after data normalization and Louvain clustering. (**B**) Top 20 differentially expressed genes (DEG), in old vs. young samples. All cells from all cell types are used in this test. Wilcoxon test, top 20 DEGs by logFC. (**C**) Expression of PDK4 and IGFNl in young and old samples.

Following quality control, pre-processing, and alignment, we generated 143,051 transcriptomes from individual nuclei (93,406 old, 49,645 young). After normalization and clustering, we did not observe any batch effect between the samples (Supplementary Figure 3). Uniform Manifold Approximation and Projection (UMAP) analyses revealed 7 distinct clusters, each corresponding to a unique cell type. We assigned cell identity to specific clusters using known markers for type II fibers, type I fibers, fibro-adipogenic progenitors (FAPs), satellite cells (SCs), smooth muscle cells (SMC), endothelial cells (EC) and immune cells (macrophages, T/NK cells) (Figure 2A, Supplementary Figure 4, Supplementary Table 3).

After normalizing to total myonuclei (type I + II), we then calculated the proportion of type I (slow) and type II (fast) fibers per subject that varied among the samples (Table 1). In an alternative normalization procedure, we determined the proportion of fiber types based on nuclei from all other cell types (Table 1). Regardless of normalization procedure, quantitation of fiber types within individuals agreed with values reported in the literature using histological staining (Table 1) [39]. Similarly, we calculated the proportions of each major cell type between younger and older subjects. There was no significant difference in enumeration of nuclei associated with fiber types with age, FAPs, or macrophages. However, satellite cells, endothelial cells and smooth muscle cells were all significantly reduced with age (Table 1). Nuclei identified as derived from SCs in younger subjects comprised 5% of the nuclei population, consistent with prior reports, while older subjects had only 2% SC’s, consistent with reports of loss of this muscle specific stem cell with age [40–42].

**Table:** Table 1A. Normalized by total myonuclei (type I and II).

	**Old *N* = 11**	**Young *N* = 6**	***P***
**Slow skeletal fibers**	0.44 (0.16)	0.34 (0.13)	0.2
**Fast Skeletal fibers**	0.56 (0.16)	0.66 (0.13)	0.2
**FAPs**	0.13 (0.08)	0.15 (0.08)	0.732
**EC**	0.04 (0.02)	0.10 (0.03)	0.005
**Macrophages (T/N.K cells)**	0.05 (0.04)	0.06 (0.03)	0.877
**SCs**	0.02 (0.01)	0.05 (0.03)	0.034
**Smooth.MC**	0.02 (0.01)	0.06 (0.02)	0.001

**Table:** Table 1B. Normalized by total nuclei.

	**Old *N* = 11**	**Young *N* = 6**	***P***
**Slow skeletal fibers**	0.34 (0.12)	0.24 (0.08)	0.057
**Fast skeletal fibers**	0.45 (0.15)	0.47 (0.11)	0.757
**FAPs**	0.10 (0.05)	0.10 (0.05)	0.956
**EC**	0.03 (0.01)	0.07 (0.02)	0.002
**Macrophages (T/N.K cells)**	0.04 (0.03)	0.04 (0.02)	0.912
**SCs**	0.02 (0.01)	0.04 (0.02)	0.033
**Smooth.MC**	0.02 (0.01)	0.04 (0.01)	0.002

Overall, these findings are consistent with previous muscle single nuclei studies in rodents and humans [19, 21, 22, 24, 43] not focused on aging. The fast (*MYH1*/2) and slow (*MYH7*) myonuclei populations were present in our study, but *MYH4* was not a marker in our dataset. FAPs, SATs, ECs, SMCs, T/NK and macrophages were similarly present in our dataset. We did not find a population of tenocytes (*SCX*), or myotendinous cells (*COL22A1*), previously reported by Dos Santos et al. [21]. Similarly, we did not find a population corresponding to the neuro-muscular junction (NMJ) (*CHRNE, MUSK*) in our dataset [44]. Functional denervation of individual motor units has been proposed as a cause for sarcopenia in rats [45]. Further, serum levels of C-terminal agrin were reported to be associated with sarcopenia, and as an indicator of instability and loss of the NMJ [46]. We tested this hypothesis by assessing the levels of agrin in the serum from individuals in our cohort, but we could not identify a statistically significant difference between young versus older subjects.

### *PDK4* is upregulated and *IGFN1* is downregulated with age in all cell types

We next examined broad changes between young and old samples by pooling all cell types. Notable were an upregulation of *PDK4* and downregulation of *IGFN1* expression. *IGFN1* was expressed in 49% of young sample nuclei, compared to 5% of old sample nuclei. *PDK4* was expressed in 15% of young nuclei, and 43% of old sample nuclei (Figure 2B, 2C). We next compared the aging signatures obtained from our bulk study to this pooled single nucleus aging signature. Both bulk and single nuclei shared several commonly dysregulated genes, with marked *IGFN1* downregulation and *UNC13C upregulation,* using both methods (Supplementary Figure 2D).

**Figure 3:**
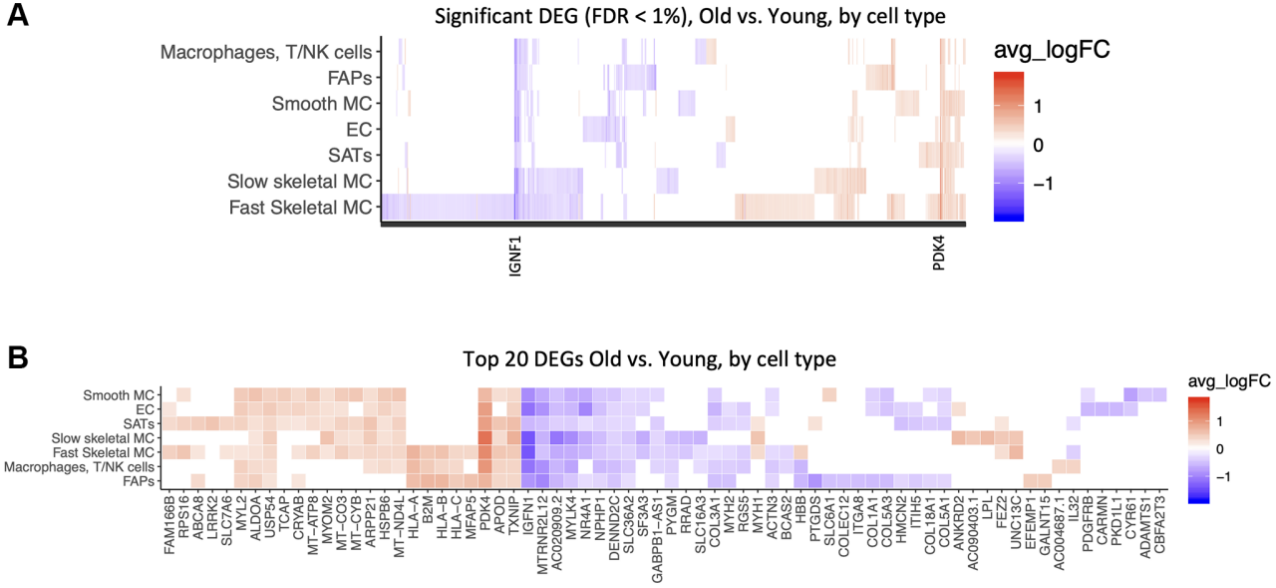
**Common and cell-type specific gene expression changes with age.** Significant differentially expressed genes (DEG) in old versus young samples. A Wilcoxon test was performed for each gene in each cell type between samples, with a logFold-Change (logFC) threshold of .25, and False-Discovery Rate (FDR) <1%. Red is upregulated with age, blue is downregulated. (**A**) All DEGs are shown by cell type. (**B**) Top 20 DEGs are shown by cell type, ranked by absolute logFC.

We next examined cell-type specific changes with age. We identified 1,343 significant differentially expressed genes (DEGs), at a false discovery rate (FDR) of 1%. Some of these changes were common to many cell types, including those with altered *PDK4* and *IGFN1* expression, but most were cell type specific. Of note, the transcriptomes that changed the most with age were from fast skeletal muscle fibers (Figure 3A). HLA genes (*HLA-A, HLA-B, HLA-C, B2M*) were upregulated in FAPs, immune cells and Fast Skeletal muscle. Mitochondrial genes (*MT-ATP8, MT-CO3, MT-CYB)* were upregulated in smooth muscle, satellite cells, and endothelial cells*. PDK4, APOD, TXNIP* were upregulated in most cell types. *IGNF1, MTRNR2L12, MYLK4, NR4A1* declined in expression with age in most cell types. (Figure 3B, Supplementary Table 2) It is well recognized that different muscle fiber types change dynamically with age. Among recognized fiber type markers [47, 48], *MYH1* (fast type 2×), *MYH2* (fast type 2A), *MYH7* (slow type I fibers) and *MYH7B* (slow type I fibers) were present in our dataset, and *MYH2* expression significantly declined with age, consistent with previously reported type II fiber atrophy [49].

**Figure 4:**
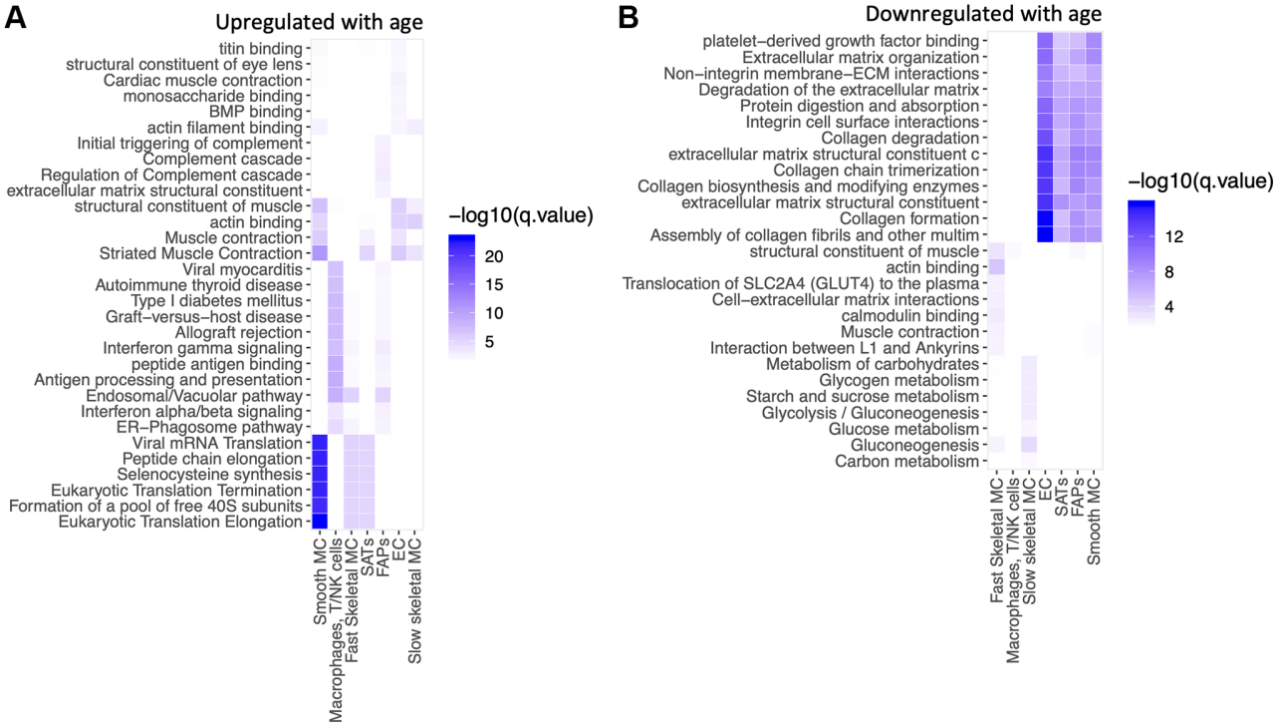
**mRNA translation, gamma interferon and complement cascade are upregulated in selective cell types with aging.** Pathway analysis of top 100 up-regulated and top 100 down-regulated genes with age in each cell type. GO, KEGG, Reactome pathways were queried. Over-representation was assessed using a hyper-geometric test at FDR 1%. (**A**) Upregulated with age. (**B**) Downregulated with age.

We then performed a pathway analysis of the age-related changes within each cell type (Figure 4A, 4B). Several pathways related to mRNA translation were upregulated with age in SMCs, SCs and fast SMC (Figure 4A). Antigen presentation, gamma interferon responses and complement cascades were upregulated in immune cells (Figure 4A). Muscle contraction pathways were upregulated in slow SMCs (Figure 4A). Conversely, pathways related to collagen and extracellular matrix (ECM) declined with age in ECs, SCs, FAPs and SMCs (Figure 4B). Glucose metabolism was downregulated in SMCs (Figure 4B). Table 2 shows a summary of differentially modulated genes involved in these pathways. Mitochondrial counts are frequently used as a proxy for quality control in single nuclei library preparation [50]. Therefore, we also tested the percentage of counts coming from the mitochondrial genome in young and old samples. For young samples 0.47% (0.27%–0.89%) of counts belonged to the mitochondrial genome, for old samples this number was 0.71% (0.37%–1.3%) (*p* < 2.2e10-16, Wilcoxon). This finding suggests a small but significant increase in mitochondrial counts from young to old samples, potentially implying more cell death, or “leakiness” in our aged biopsies.

**Table 2:** Summary of pathways dysregulated with age, including implicated genes.

**Summary (Up)**	**Summary (Down)**
Translation:	Collagen/ECM:
*RPLP0/RPL10/RPL37A/RPS16/RPL13A/RPL21/…*	COL3A1/COL5A3/COL6A2/COL4A1/COL15A1/COL1A2/…
Antigen presentation/Interferon gamma:	Glucose metabolism:
*HLA-A/HLA-B/B2M/HLA-C/HLA-DRB1/HLA-DRA/HLA-F/…*	TPI1/GPI/ENO3/FBP2/GOT1
Complement cascade:	
*C1S/C3/C1R*	
Muscle contraction:	
*MYL2/TCAP/TNNC2/TNNT1/TNNC1/ACTN2/TNNI1*	

### Identification of a small population of *CDKN1A/MYH8/COL19A1/LRRK2/EDA2R*+ cells in aged muscle consistent with senescence

To investigate how muscle cell composition changes with age, we determined the frequencies of different subtypes within each sample. Our approach bypasses laborious counting of histological sections to enumerate distinct cell types, and enzymatic digestions followed by isolation of cells with known specific markers using flow cytometry. It also has the capability to define novel cell subtypes that may drive pathology.

**Figure 5:**
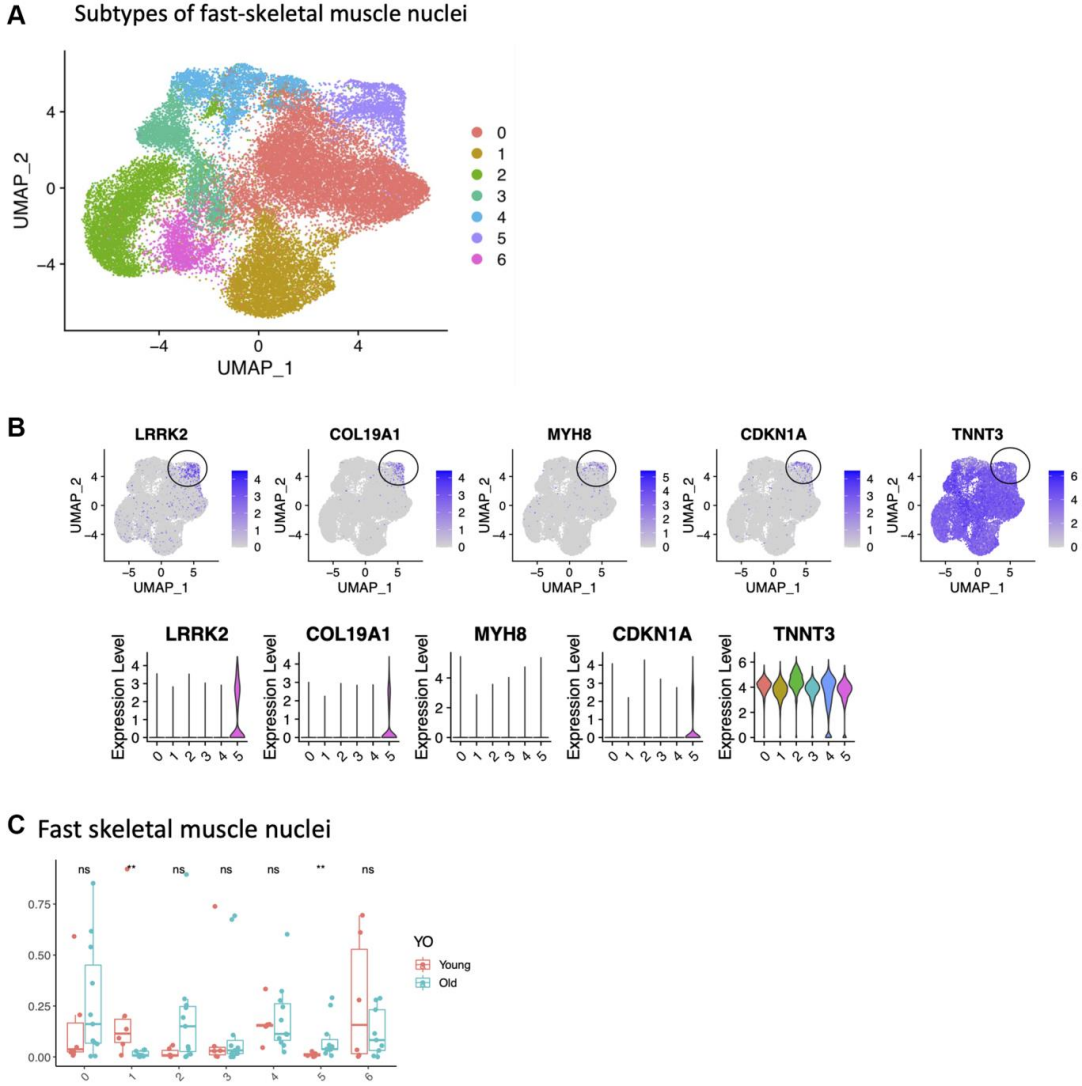
**Identification of a small population of senescent cells in the fast skeletal muscle.** (**A**) Subtypes of fast-skeletal muscle cells (UMAP, all samples). (**B**) Cluster 5 is circled, with expression of LRRK2, CDKN1A, MYH8, COL19A1 and TNNT3. (**C**) Difference in proportions between young and old for all subtypes. Significance of the *t*-test between young and old is shown at the top of 5C.

We first examined FAPs cells identified by snuc-Seq profiling. One cluster was largely *PDGFRA*+, but in the other we observed expression of the muscle contraction gene Tryadin (*TRDN*) and skeletal muscle ryanodine receptor (*RYR1*) gene. Tryadin and *RYR1* are known to interact, and are involved in muscle contraction [51]. We speculate that these nuclei are derived from cells attached to or contaminated by muscle fiber markers, since these markers have not yet been reported in FAPs. However, the frequency of neither subtype changed with age (Supplementary Figure 5A). We also identified infiltrating immune cells, including T/natural killer (NK) cells, macrophages and mast cells. There were no changes with age in the frequency of these immune cells (Supplementary Figure 5B). For *CD34*^+^ nuclei, we identified two main cell types. One cluster expressed *PDGFRB*, and was consistent with vascular smooth muscle cells. There was no change in the frequency of these cells with age (Supplementary Figure 5C). Consistent with prior reports, we observed an ~ 20–40% decrease in satellite cell frequency with age when normalized to total myonuclei (type I + II fibers) (Table 1). However, within SCs, which were Pax7^+^, we observed several distinct subclusters, possibly defining functional subgroups for this cell type. One subcluster expressed *LGR5* and *MYH7B,* which in a recent report represents a subset of activated SCs that contribute to muscle regeneration [52] (Supplementary Figure 6). Lastly, we tested whether the transcriptomes derived from fast skeletal muscle fibers revealed more than one subtype. All clusters were present in equal proportions between young versus old samples, with one exception. This unique cluster contained *CDKN1A/MYH8/COL19A1/LRRK2/EDA2R*^+^ cells and was present only in samples from older adults (Figure 5). Interestingly 4 of these genes (C*DKN1A, MYH8, COL19A1, EDA2R)* were also among the top upregulated genes with aging in the bulk RNASeq study. A recent single nuclei study in mice also identified a subset of senescent myonuclei expressing CDKN1A and EDA2R in old muscle [53], implying this subpopulation is conserved between mice and humans. Type II fibers are known to atrophy with age [49], but the role of these specific genes in age-related atrophy remains to be determined.

### Spatial transcriptomics captures aging muscle fiber reorganization, and validates RNA sequencing

**Figure 6:**
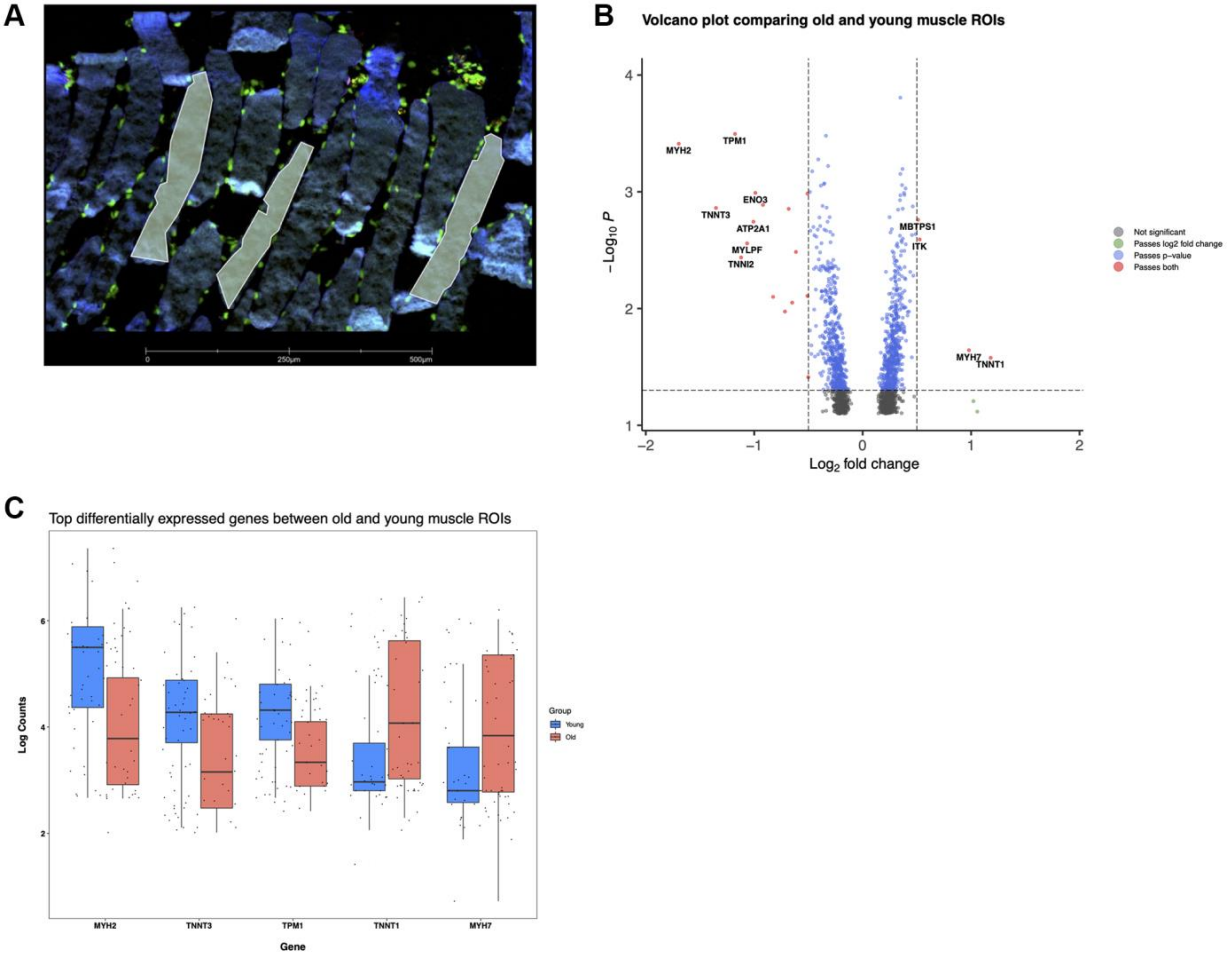
**Reorganization of muscle fibers with age revealed by spatial transcriptomics.** (**A**) Young muscle fibers, several ROls are shown in yellow delineating individual sections of distinct fibers. Desmin (blue), Syto83 (green), aSMA (yellow), CD68 (red). (**B**) Differentially expressed genes in old versus young spatial profiled muscle. (**C**) log (counts) of top differentially expressed genes.

As an alternative approach to validate our snuc-Seq results, we performed spatial transcriptomic profiling of multiple paraffin embedded human skeletal muscle biopsies (4 young and 3 old subject samples) using a GeoMx Digital Spatial Profiler (DSP, nanoString Figures 4, 5). Typically, muscle is embedded for histology in a cross-sectional fashion, showing multiple characteristics of fiber structure (e.g., fiber cross-sectional area). However, in this orientation, samples are incompatible with DSP technology applied to single fibers due to limitations on probe capture from minimum surface area/region of interest. Therefore, we embedded fibers in a longitudinal profile, permitting investigations by DSP for single fibers, as this approach increased the available area interrogated per individual fiber relative to cross-sectionally embedded samples. Tissue sections were stained with the nuclear marker DAPI to mark nuclei, and with the muscle specific marker Desmin (Figure 6A). Regions of interest (ROIs) were chosen preferentially within longitudinal sections, enabling profiling to be performed within distinct individual muscle fibers. Whole transcriptome spatial profiling was carried out in 80 total ROIs (40 young, 40 old (Figure 6B)). Comparisons of gene expression between young versus old revealed 1041 significantly differentially expressed genes (DEGs), of which 530 were upregulated and 511 downregulated. Top upregulated genes include slow skeletal type troponin *TNNT1* and the myosin heavy chain *MYH7*, whereas *MYH2* and fast skeletal type troponin *TNNT3* were downregulated (Figure 6C). This finding indicates selective atrophy from fast (type 2) to slow (type 1) twitch fibers, confirming previous findings [54–56], and validates spatial profiling as a tool to investigate age-related loss of function. We also identified an upregulation of *ITK*, a positive regulator of the SASP factor *IL-8* [57, 58]. Together with an upregulation of proinflammatory cytokine *IL1B*, a SASP enriched cellular milieu can be inferred, implying senescence is an active component of aging skeletal muscle.

Of the total 1041 genes, 225 were also validated as DEGs in the bulk RNAseq data. Of these 225, 11 genes are further shared with pseudo bulk-derived DEGs from the single nuclei RNA data: NT5C2, SAMD4A, LGR5 (upregulated), FBP2, NDRG2, IGFN1, COX6A2, TPI1, ALDOA, TNNC2, MYH2 (downregulated). Pathway analysis revealed inhibition of oxidative phosphorylation, glycolysis, gluconeogenesis, and *NRF2* signaling. While Sirtuin signaling was activated overall, only SIRT6 was differentially regulated, and in a negative direction as previously reported in other aging studies [59–61]. Toll-like receptor signaling was also activated in sarcopenic patients, an aging response also seen in macrophage STAT1 phosphorylation impairment and decreased PI3-kinase activity in myeloid dendritic cells [62, 63]. The *ERK/MAPK* pathway is also activated in aged ROIs in our skeletal muscle and has previously been linked to several hallmarks of aging including DNA damage, oxidative stress, and RAS signaling [64, 65].

*DDX5* was identified as the top inhibited regulator in aged samples. In a 2019 study from Fan et al. [66], *DDX5* was shown to be required for maintaining vascular smooth muscle cell homeostasis and quiescence. This RNA helicase may thus play an important role in the maintenance of healthy muscular vasculature, making it a potential therapeutic target in sarcopenic or frail patients.

### Confirming gene expression signatures from aged skeletal muscle in cultured senescent myoblasts

**Figure 7:**
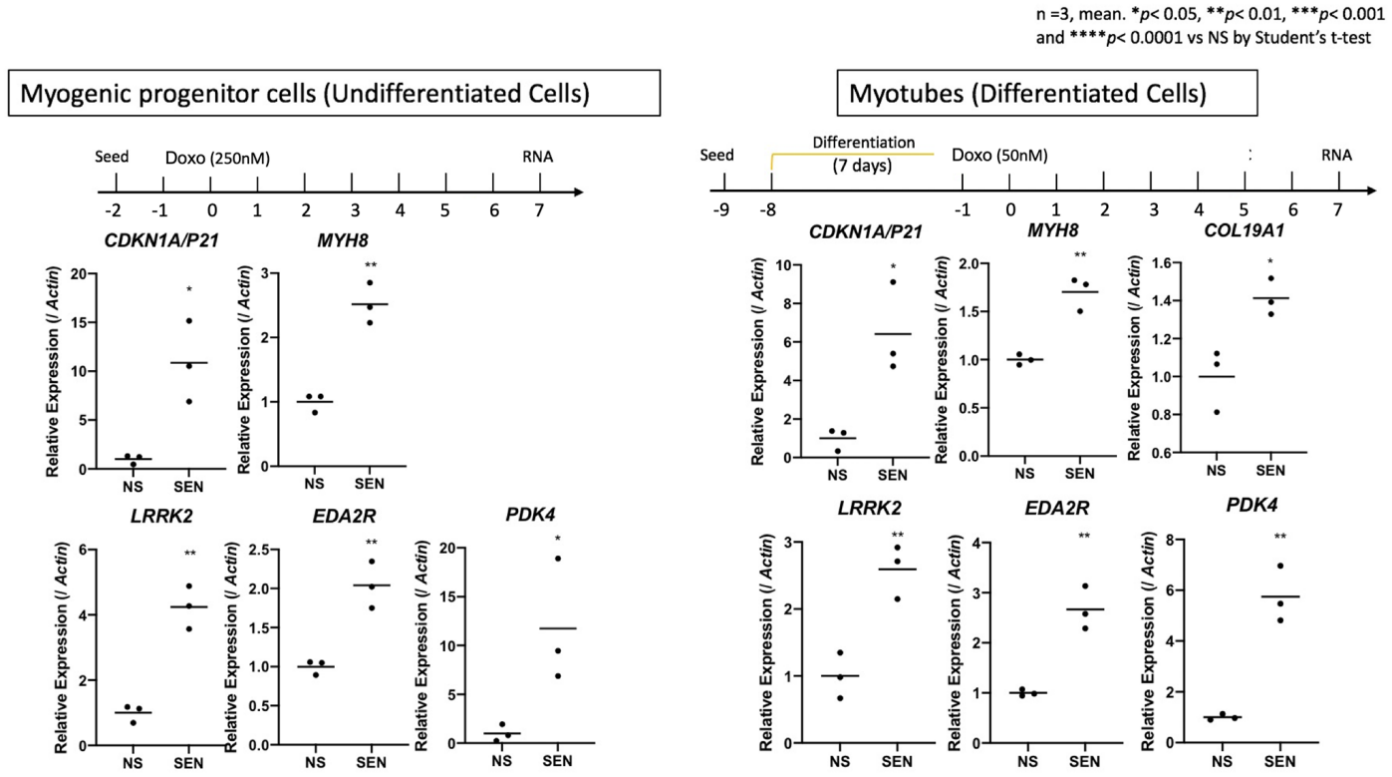
**Validation of senescent markers in cultured human muscle cells.** Quantitative PCR (qPCR) of CDKN1A, MYH8, COL19A1, LRRK2, EDA2R and PDK4 after 7 days of incubation in senescent vs. non-senescent cells. Senescence was induced using Doxorubicin in a cell line of Myogenic Progenitor cells (un-differentiated cells, left) and Myotubes (differentiated HSMMs, right). Expression is shown relative to Actin. 3 replicates in each condition/gene.

We wished to validate our findings regarding senescent cells arising with age in skeletal muscle. Therefore we tested the hypothesis that induced senescence in a muscle cell culture model would recapitulate some of the gene signatures we identified in *CDKN1A*-expressing populations of nuclei detected in old muscle samples. We induced senescence in cultured primary human skeletal muscle myoblast cells (HSMMs), comprised of both myogenic progenitor cells and myotubes, using the genotoxic agent doxorubicin, an increasingly used senescence inducer [67–69]. After 7 days post doxorubicin exposure, we determined expression of the following genes: *CDKN1A, MYH8, COL19A1, LRRK2, EDA2R* and *PDK4* in senescent versus non-senescent cells by quantitative PCR (qPCR). In both cell types, all target genes were significantly upregulated in the senescent compared to non-senescent cells (Figure 7). These results suggest that cultured senescent cells derived from muscle exhibit some of the same markers we identified in nuclei derived from muscle biopsies of older frail individuals. We recently reported the small heat shock protein *CRYAB* as a critical mediator of senescence in multiple cell types from both mouse and human tissues including muscle [68]. CRYAB has been previously reported to increase with age, and specifically aggregate within human skeletal muscle [70]. We therefore measured CRYAB gene expression in individual nuclei via snuc-Seq, and determined small but significant increases in CRYAB gene expression in multiple cell types isolated from young versus old skeletal muscle (Supplementary Figure 7), consistent with both our cultured myoblast gene expression studies, and increased senescence with age within subpopulations of cell types comprising skeletal muscle.

## DISCUSSION

In this study, we determined changes in gene expression due to aging and frailty in multiple distinct cell types derived from human skeletal muscle. We analyzed and verified senescent gene expression signatures derived from bulk RNA-seq and a cell type-specific approach using single nuclei sequencing, digital spatial profiling, and cultured human skeletal muscle myoblasts.

At the bulk RNA-seq level, the top markers that increased with age and frailty were *MYH8, COL19A1* and *CDKN1A*, in agreement with previous studies showing increased expression of *CDKN1A* (p21) and *MYH8* in muscle biopsies from older individuals [12, 71]. *MYH8* encodes the developmental protein myosin-heavy chain 8 (MYH8) [48]. It is transiently expressed during embryonic development, but expression declines shortly after birth. It is also necessary for muscle regeneration after injury [72]. The age-related rise in MYH8 expression, given its reported association with development and injury repair, suggests that aged muscle tissue chronically attempts to repair age-related damage. Further, a mutation in *MYH8* is associated with severe congenital muscle contractile disorders (distal arthrogryposis syndromes) [48]. Thus, nuclei expressing *MYH8* in aged skeletal muscle may indicate a response to age-related macromolecular damage or may represent a unique marker of muscle dysfunction. Interestingly, one prior report determined that HIV patients showed signs of premature muscle aging with similar gene expression signatures [71]. We determined that several markers previously reported to be upregulated in both rat and human muscle with age were also senescent markers: cyclin-dependent kinase inhibitors *CDKN2B* (p15^Ink4b^) and *CDKN1A* (p21), and the p53-dependent ectodysplasin A2 receptor (*EDA2R*) [73, 74].

At the single nuclei level, we report broad upregulation of *PDK4* and downregulation of *IGNF1* expression in muscle from older individuals. *IGFN1* encodes the immunoglobulin like and fibronectin type III domain-containing protein (IGFN1), and is required for myoblast fusion and differentiation [75]. *PDK4* encodes pyruvate dehydrogenase kinase 4 and is overexpressed in skeletal muscle in patients with type 2 diabetes, fasting, and immediately post exercise [76–79]. We also detected an upregulation of several HLA genes (*HLA-A, HLA-B, HLA-C*), including the HLA gene *B2M*, a putative pro-aging factor in plasma that could be detrimental for cognition [80]. This finding is compatible with increased gamma interferon signaling with age [81], and supports the long standing hypothesis that inflammation is a significant component of aging [82] and muscle dysfunction [83]. Interestingly, *CDKN2A* (p16^Ink4a^), a classical senescence marker was not observed to be upregulated with age in bulk or single nuclei results. *CDKN2A* is alternatively spliced and is best detected from 10× single cell libraries using 5’ library preparation, to increase the likelihood of detecting the alternatively spliced p16^Ink4a^ transcript. Even though we used the 5’ library approach, we still did not detect increased levels of p16^Ink4a^ with age.

Gene expression of *MYH2* (a type II fiber-specific gene) declined with age consistent with prior data [10]. We also quantitated a global decrease in the proportion of satellite cells in aged muscle, consistent with a loss of muscle satellite cells, which is well established in the literature [41, 49, 84]. Further, we identified a small population of *CDKN1A/MYH8/COL19A1/LRRK2*^+^ nuclei present only in muscle from older individuals. The expression profile of this population is consistent with that of senescent myonuclei, and may thus be targeted by senolytic compounds to improve muscle function [85]. We also confirmed several previously reported major aging pathways that are dysregulated in aged muscle. Collagen pathways declined with age, consistent with prior reports of decreased collagen production being a major phenotype of aging [86]. Translation pathways also increased with age. Interestingly, mTOR, a gene strongly implicated in mediating degenerative changes with age, also regulates mRNA translation [87]. Hence, our results also reinforce existing literature implicating fibrosis and mTOR dysregulation in aging skeletal muscle [88, 89].

Recent studies have explored changes in aging muscle at the single cell level. One study found a chronically inflamed state in aged muscle, using a new spatial method called CODEX [90]. Another study focused on senescent cells in old mouse muscle, identified a population of p21-expressing myonuclei exhibiting senescent phenotypes, consistent with our results [53]. Lastly, a study probing intercostal muscle from young and old humans, using single cell and single nuclei RNA-seq, found inflammation, fiber typing changes and microenvironment alterations, to be distinct mechanisms driving muscle aging [91].

We determined an association between differential gene expression at the bulk RNA level and clinical phenotypes. For example, increased *IGFN1* expression was associated with improved performance (‘Timed up and Go’). Accordingly, we hypothesize that *IGFN1* has a beneficial role in aged muscle. In further support of this hypothesis, *IGFN1* mRNA was shown to increase in elderly muscle after an exercise training regimen [92]. In contrast, low levels of *COL19A1* (Collagen XIX Alpha 1) expression were also associated with improved performance in the isometric knee extension torque (‘Biodex’), which may indicate that increased *COL19A1* expression relative to young individuals is detrimental to muscle function. High *COL19A1* expression also correlates with poor prognosis in Amyotrophic Lateral Sclerosis (ALS) [93]. The fact that some transcripts, regardless of age, are associated with muscle performance could indicate that maintaining these genes at their appropriate level may help counteract muscle functional decline with age.

Mature differentiated muscle is challenging to study because muscle fibers are difficult to isolate, and many nuclei operate in concert along the fiber with yet to be elucidated zones of influence. One approach is to dissect out individual muscle fibers and characterize them individually [25]. An alternative approach is to use single nuclei sequencing to capture gene expression from all nuclei in the fibers and all cells captured from a fresh muscle biopsy. In some cases, results from single nuclei can be concordant with bulk gene expression [26]. The newly emerging technique of digital spatial profiling, for identifying gene expression within fixed or frozen tissue, offers great potential for localizing differentially expressed genes and proteins in the context of tissue micro-architecture. Here we applied this technique to young and old tissue sections of fixed skeletal muscle and confirmed a number of differentially expressed genes with *in-situ* localization to the messages present within individual muscle fibers. In addition, using our overall workflow, we were able to enumerate changes in frequency of all cell types with age, providing a new gold standard methodology for understanding cell composition changes with age.

Our study provides several insights into human skeletal muscle aging. It revealed remarkable heterogeneity in gene expression patterns in this tissue, both within individual fibers and between fibers and other cell types comprising skeletal muscle. It also provides insights into mechanisms of muscle aging and frailty. We confirmed several of these markers of cellular senescence in cultured human senescent cells, validating our hypothesis that senescence is indeed contributing to the decline in function of aged human skeletal muscle. Using the array of technologies we describe here, we show that higher inflammation by pathway analysis, stem cell exhaustion shown by decrease in satellite cells, shifts in fiber typing, and increased markers of senescence all play a role in muscle aging and frailty.

## MATERIALS AND METHODS

### Subject recruitment

We recruited adults between the ages of 18–30 and 65–85 years old. Individuals were recruited from the community using advertisements in newspapers, at grocery stores, community centers and McMaster University. In addition, participants met with the study coordinators and had the trial explained to them.

### Inclusion criteria

We recruited relatively inactive younger men and women (<1 hour of formal exercise/week) who were in the overweight category (body mass index or BMI 25–29.9 kg/m^2^). The non-sarcopenic older male participants had a BMI of <30 kg/m^2^, muscle mass index of >7.23 kg/m^2^, and a 4-meter walk test of >0.8 m/s. Subjects in the older adult sarcopenia group had a BMI of <30 kg/m^2^, a muscle mass index of 8.51–10.75 kg/m^2^, and a 4-meter walk test of <0.8 m/s.

### Exclusion criteria

Medical conditions that precluded participation were diabetes mellitus (requiring more than one anti-diabetic drug), recent myocardial infarction (<6 months ago), hypertension (requiring more than two medications), congestive heart failure (requiring more than one medication), previous stroke with residual hemiparesis, renal disease (creatinine >140), liver disease, musculoskeletal injury affecting exercise tolerance, musculoskeletal disorder (other than age-related SM wasting), severe osteoporosis, severe osteoarthritis, severe peripheral neuropathy, chronic obstructive pulmonary disease (FVC or FEV1 <70% of age-predicted mean value), asthma (requiring more than two medications), gastrointestinal disease, infectious disease, inability to consent, lactose intolerance/dairy protein allergy, and the use of medications affecting protein metabolism (for example, corticosteroids). Lifestyle-associated behaviors that precluded enrollment were smoking, veganism, recent weight loss or gain (<3-month period prior to the study), PA levels exceeding the minimal recommendations (150 min/week), and intake of supplements that affect musculoskeletal metabolism (e.g., whey, casein, calcium, creatine monohydrate, vitamin D, and omega-3 fatty acids).

### Subject classification

Subjects were classified into young adults, non-sarcopenic old adults and sarcopenic old adults according to the criteria in Supplementary Table 2.

### Biopsy collection

Participants arrived in the morning in a fasted state (10–12 hr) and rested in the supine position for 10 minutes. A muscle biopsy was then taken from the vastus lateralis using local anesthetic as previously described [32].

### RNA extractions from muscle biopsies for bulk analysis

Muscle biopsies were homogenized using a mortar and pestle with liquid nitrogen. RNA was extracted from the powdered samples using the RNeasy Fibrous Tissue Mini Kit (Qiagen) and the QIAcube automatic processor (Qiagen). Integrity and concentration of the RNA was assessed using the Tapestation 4200 (Agilent Technologies), with the cut-off for acceptable integrity being an RNA integrity number (RIN) >7. Batch-tag-seq libraries were then produced from the RNA and run on the HiSeq 4000 by the DNA Technologies Core at U.C. Davis.

### Single nuclei RNA extraction and processing

Nuclei were isolated from the biopsies using the Singulator instrument (S2 Genomics). The instrument was primed with cold nuclei isolation and storage buffers (S2 Genomics) and the biopsy was loaded into the cartridge and covered with the 19.7 mm grinding cap. The “Nuclei_All_Tissues” protocol was used to isolate nuclei, after which the nuclei were centrifuged for 5 minutes and resuspended in buffer. The nuclei were counted using a Countess II Automated Cell Counter (Invitrogen), centrifuged again for 5 minutes, and resuspended at the proper concentration for use in the Chromium Single Cell 5’ Library and Gel Bead Kit v1 (10× Genomics). Samples were processed using the Chromium Single Cell A Chip Kit and Chromium Controller (10× Genomics). Quality control was performed on the Tapestation 4200 (Agilent Technologies). Libraries were sequenced in one lane of a NovaSeqS4 by the U.C. Davis Genomics Core.

### Bulk RNAseq data analysis

Reads were aligned to the human genome using the STAR aligner and GRCh38 as the reference genome. Counts were computed using the featureCounts function in the subread software. Genes with a total count of less than 10 were removed from the analysis. PCA of differentially expressed genes were derived by the DESeq2 library in the R software, with absolute log Fold Change (logFC) >1.5 and False-Discovery Rate (FDR) <5%. Supplementary Table 3 shows QC details for libraries prepared for each sample. As a convention, anytime we refer to “A vs. B”, genes with a positive fold-change are upregulated in “B”.

### Single-nuclei 5’ RNAseq data analysis

Reads were mapped to the human genome using Cell Ranger (10× Genomics), and the GRCh38 genome reference. Cells were removed if they expressed <200 unique genes. Genes not detected in any cell were removed from subsequent analysis. One sample was discarded due to low quality. Samples in this study averaged less than 1% mitochondrial counts per sample, but counts were higher in the old samples (*p* < 10^–16). Read count normalization, variable feature detection (nfeatures = 2000), scaling, UMAP (ndim = 10), and differential expression were computed as described in the Seurat package [94]. Clustering was performed by the Louvain algorithm (resolution = 0.2). No batch effect was observed. Cell types were characterized by a combination of known markers and de novo cluster markers (Table 1).

### Pathway analysis

To derive the pathways containing differentially regulated genes, we performed a hyper-geometric test to assess over-representation. We used the clusterProfiler R package on the database Gene-Ontology (GO), Kyoto Encyclopedia of Genes and Genomes (KEGG). Gene set enrichment analysis was performed [95], using log Fold-Change as ranking metric.

### Spatial transcriptomics

Paraffin embedded human skeletal muscle biopsies (4 young, 3 old) were profiled using the GeoMx Digital Spatial Profiler (nanoString), as previously described [96]. The standard GeoMx workflow was employed [96] 5 μm tissue sections were cut from the fresh-frozen tissues, and mounted on superfrost + glass slides and used within a week of cutting. Sections were then fixed in 10% NBF for overnight, then antigen retrieval followed by a proteinase K digestion to make available RNA within the tissue (1 mg/ml). GeoMx Hu WT probes (>18,000 protein coding genes) were then incubated overnight in a humidified chamber at 37°C to allow probes to anneal to expressed genes in the tissue. Morphology markers were then applied to the tissue for 1 hour in a humidity chamber; we used a directly conjugated antibody (FITC-525 nM) against desmin (for fiber detection), and for nuclei we used Syto83 (Cy3/568 nMM). Slides containing probes and morphology markers were then loaded into the DSP, and polygonal regions of interest within individual fibers were then drawn on each sample, with up to 4 tissue sections/slide, for a total of eighty ROI’s across the 7 samples. ROI counts were normalized by area. Then, PCA, differential gene expression testing was performed using a linear model on each gene, comparing ROIs from young and old.

### Cell culture

Human skeletal muscle myoblasts (HSMMs; Lonza) were maintained at 37°C, 3% O_2_ and 5% CO_2_ in skeletal muscle growth media (SkGM2; Lonza). Differentiation was induced by replacing SkGM2 media with DMEM supplemented with 2% horse serum. For senescence induction, cells were treated with 50 nM (differentiated cells) or 250 nM (un-differentiated cells) of doxorubicin (Doxo) for 24 hours and then cultured for 7 days.

### qRT-PCR

Total RNA was isolated from HSMMs 7 days after Doxorubicin treatment using the RNeasy mini kit (Qiagen), and reverse-transcribed using the PrimeScript RT reagent kit (TAKARA Bio Inc.). Expression levels of the genes of interest were measured by real-time quantitative PCR using a CFX-384 instrument (BioRad). The sequences of the primer pairs are indicated as shown below. The amount of mRNA was normalized to that of β-ACTIN mRNA.

### Primers

**Table:** 

**Gene**	**Forward (5′–3′)**	**Reverse (5′–3′)**
CDKN1A/P21	AGTGGAATTAGCCCTCAGCA	CATGGTCCCTGGGTTCTTC
COL19A1	CGGCTGATGCAGTTTCATTTG	CCAGGTCTCCCATAAGCTTGG
LRRK2	ACGCAGCGAGCATTGTACCTT	GGCTTCATGGCATCAACTTCA
EDA2R	TGGACAGGAGCTATCCAAGGA	ACAGTCCCCACAGACAGCATT
PDK4	CCCGCTGTCCATGAAGCAGC	CCAATGTGGCTTGGGTTTCC
MYH8	AATGCAAGTGCTATTCCAGAGG	ACAGACAGCTTGTGTTCTTGTT
β-ACTIN	CGACAGGATGCAGAAGGAGA	CGTCATACTCCTGCTTGCTG

### Data availability

Bulk and single cell RNA-seq counts and raw data have been posted on Gene Expression Omnibus (GEO) GSE167186).

## Supplementary Materials

Supplementary Materials and Methods

Supplementary Figures

Supplementary Tables 1 and 2

Supplementary Table 3
