# Supplementary material for: Single nuclei profiling identifies cell specific markers of skeletal muscle aging, frailty, and senescence
Source: Aging (Albany NY). 2022 Dec 13;14(23):9393–422. doi: 10.18632/aging.204435 (PMC9792217; doi:10.18632/aging.204435)
Supplement: Supplementary Materials and Methods [file aging-14-204435-s001.pdf]

SUPPLEMENTARY MATERIALS AND METHODS

Physical performance assessment

Physical performance battery

A total of four functional tasks was performed once using a stopwatch that records to an accuracy of 0.01 second. For the 6-minute walk test, participants walked as fast as possible around a 20 m track for 6 minutes and distance measured to the closest meter. The standard 4-stair climb test involved participants climbing 4 stairs as fast as possible. The 5× sit-to-stand test involved participants performing a series of consecutive rising and sitting positions from a sturdy, armless plastic chair secured against a wall, with arms crossed at the chest. Finally, the chair rise-and-walk test involved starting from a seated position, standing and walking as quickly as possible in a predetermined straight line to a 9.14 m pylon, while going around the pylon, and returning to the original seated position.

Leg press 1RM

Assessment procedures for determining lower body strength using leg press exercise equipment (Cybex Eagle®, Medway, MA, USA) required participant to sit in the leg press machine with the right and left foot on the weight platform. The seat and back pad were adjusted so that feet were flat on the platform a hip-width apart, toes slightly angled out and legs parallel to each other. The interviewer then instructed the participant to grasp the handles or sides of the seat and extend their legs leaving a slight bend in the knee. Next, the participant removed the racking mechanism from the platform and grasped the handles or seat again. The participant began with a selected weight that is within their perceived ability, ~ 60 to 80% of maximum capacity. The participant lowered the platform slowly and controlled towards the chest, keeping hips and buttocks on the seat and the back flat against the back pad. Once the thighs were parallel to the platform, the participant extended the legs, pushing the weight back to the start position as hard and fast as possible. The participant was instructed to not allow hips to shift to one side, buttocks to rise or knees to move inward or outward during this exercise. The interviewer also instructed the participant to keep heels flat and not allow the knees to go beyond the toes. Once the repetitions were completed, the participant replaced the racking mechanism and exited the leg press. These procedures were adapted from those described by the National Strength and Condition Association (2008) and American College of Sports Medicine (2013).

Hand grip strength (MVIC)

Hand grip strength was measured using an isometric dynamometer (JAMAR®, Sammons, Bolingbrook, IL, USA). The grip width was adjusted to hand size, with the arm flexed at 90°C. The participant performed three 5 s efforts with a one min rest between trials.

Knee extension (MVIC)

Isometric knee extension was measured by mechanical dynamometry (Biodex System 3, Biodex Medical Systems, Shirley, NY, USA). Participants were positioned in the machine with the knee flexed at 90°C and performed 3 × 5 s maximal voluntary contractions with 30 s rests between each trial.
