# Supplementary material for: Single nuclei profiling identifies cell specific markers of skeletal muscle aging, frailty, and senescence
Source: Aging (Albany NY). 2022 Dec 13;14(23):9393–422. doi: 10.18632/aging.204435 (PMC9792217; doi:10.18632/aging.204435)
Supplement: Supplementary Figures [file aging-14-204435-s002.pdf]

SUPPLEMENTARY FIGURES

A

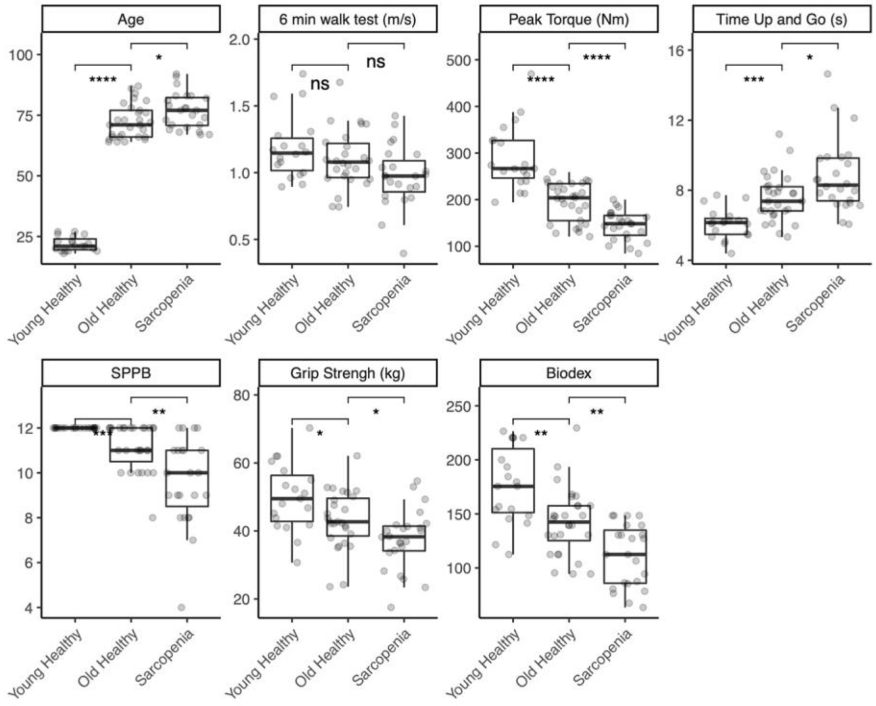

B

All subjects: BMI<36

Healthy young adults

Age 19-30, Non-sarcopenic muscle mass* or no deficit in muscle strength** or function***

Healthy old adults

Age 64-92, Non-sarcopenic muscle mass*

Mild sarcopenic old adults

Age 64-92, Sarcopenic muscle mass*

Either of sarcopenic muscle strength** or muscle function***

Severe sarcopenic old adults

Age 64-92, Sarcopenic muscle mass*

Both of sarcopenic muscle strength** and muscle function***

*ASM/Height2 < 8kg/m2

**Handgrip < 30 kg

Or Knee extension (Biodex) < 188 Nm (1.5 SD of the mean of the 19-30 year old mean)

Or Leg press < 124 kg (1.5 SD of the mean of the 19-30 year old mean)

***6 m walk test < 1 m/s

Or Timed up and go (TUG) > 12s

Or Short Physical Performance Battery < 9

Supplementary Figure 1. Clinical characteristics of the bulk cohort. (**A**) Boxplots of samples classified as young healthy, old healthy, mild sarcopenic and severe sarcopenic using the criteria shown in B. (**B**) Functional classification criteria.

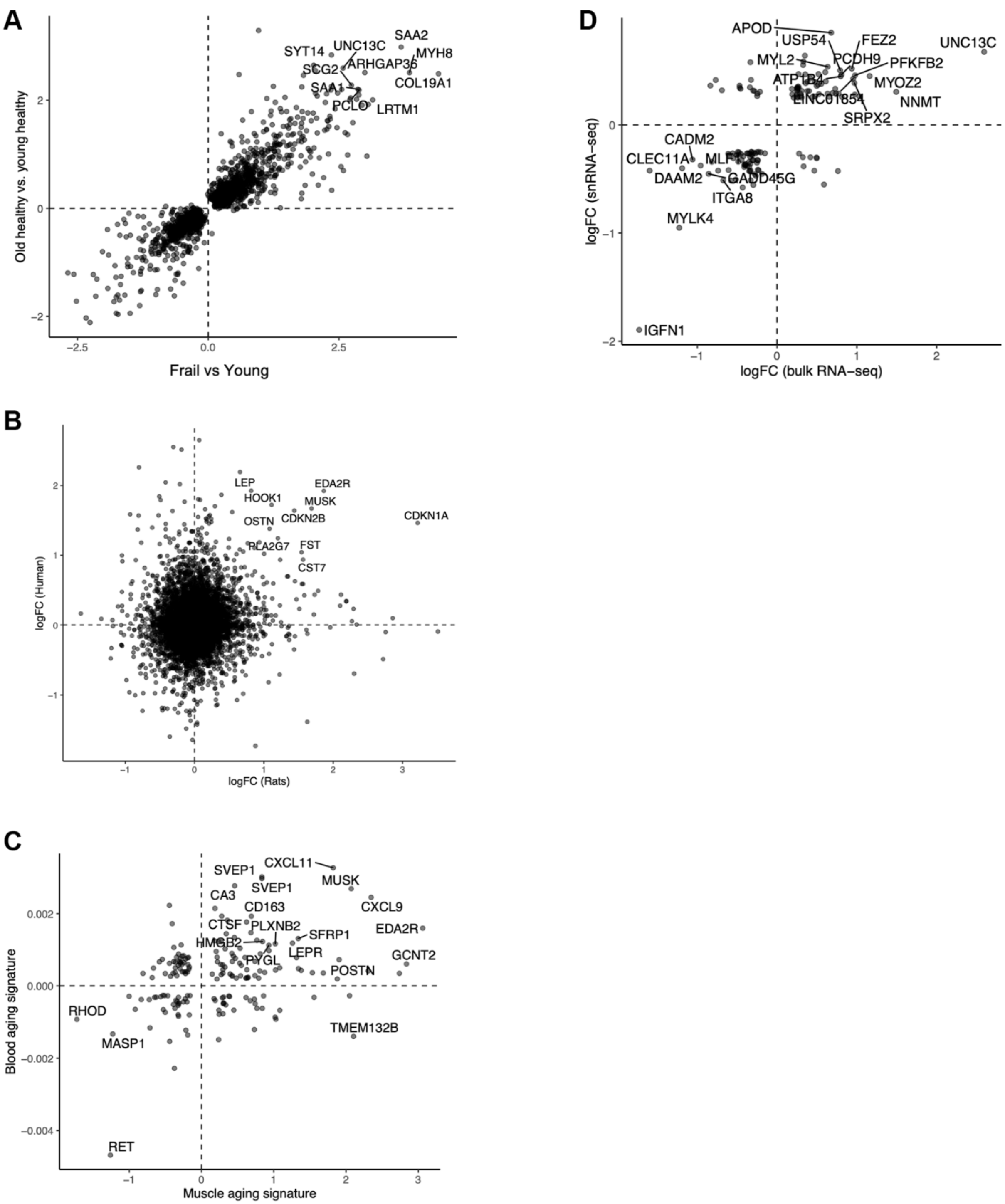

Supplementary Figure 2. (**A**) Frail vs. young; Old vs. young (R^2 = 0.9). (**B**) Conserved aging signatures in rat and human. (**C**) Comparison of muscle and blood aging signature. (**D**) Comparison of bulk and single cell aging signatures. All significantly differentially expressed genes (*p*-adj < .05) are shown. Labelled are top 20 genes with greatest logFC (X) × logFC (V).

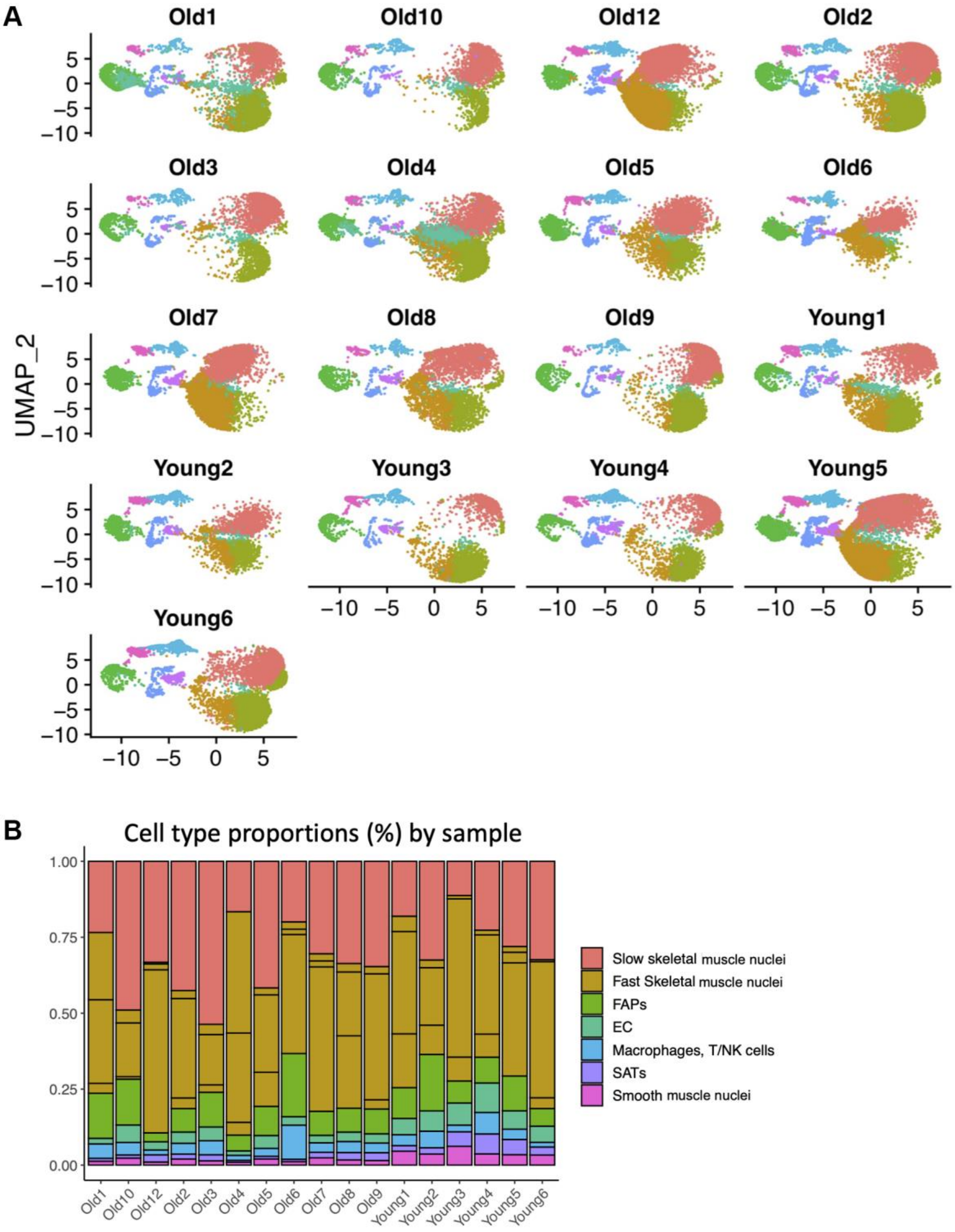

Supplementary Figure 3. (**A**) UMAP of each separate sample. (**B**) Cell type proportions in each sample.

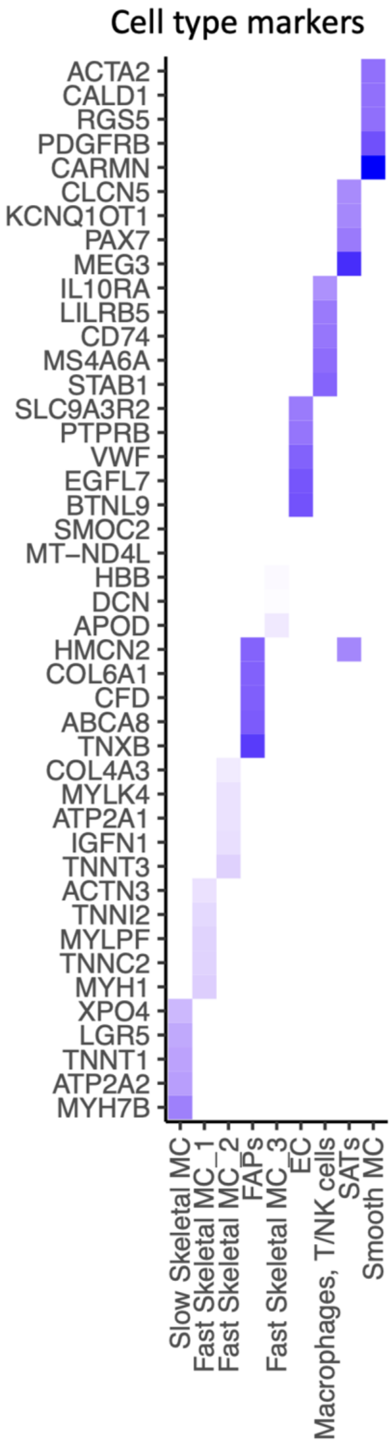

Supplementary Figure 4. Markers specific for multiple cell types in muscle. Top 5 markers for each cell type, colored by logFC. Cell type specific pathways. Top 50 DEG per cell type were fed to the GO, KEGG, Reactome databases. Over-representation was assessed using an hyper-geometric test at FDR 1%.

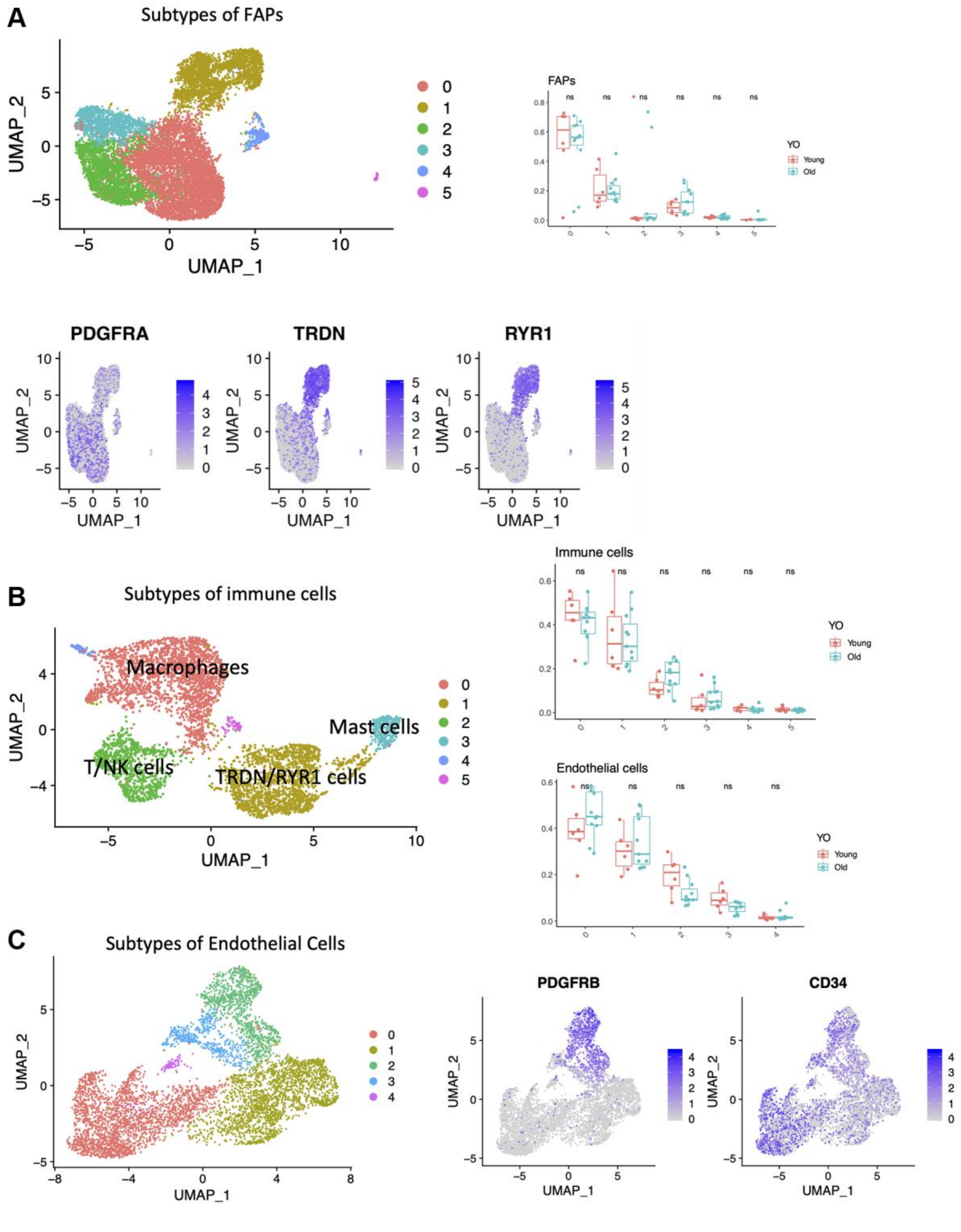

Supplementary Figure 5. (**A**–**C**) Subtypes of FAPs, immune cells, and endothelial cells. Subtypes of each cell type is shown (UMAP, all samples). Markers expressed in different subtypes. Difference in proportions between young and old for all subtypes. Significance of the *t*-test between young and old is shown at the top.

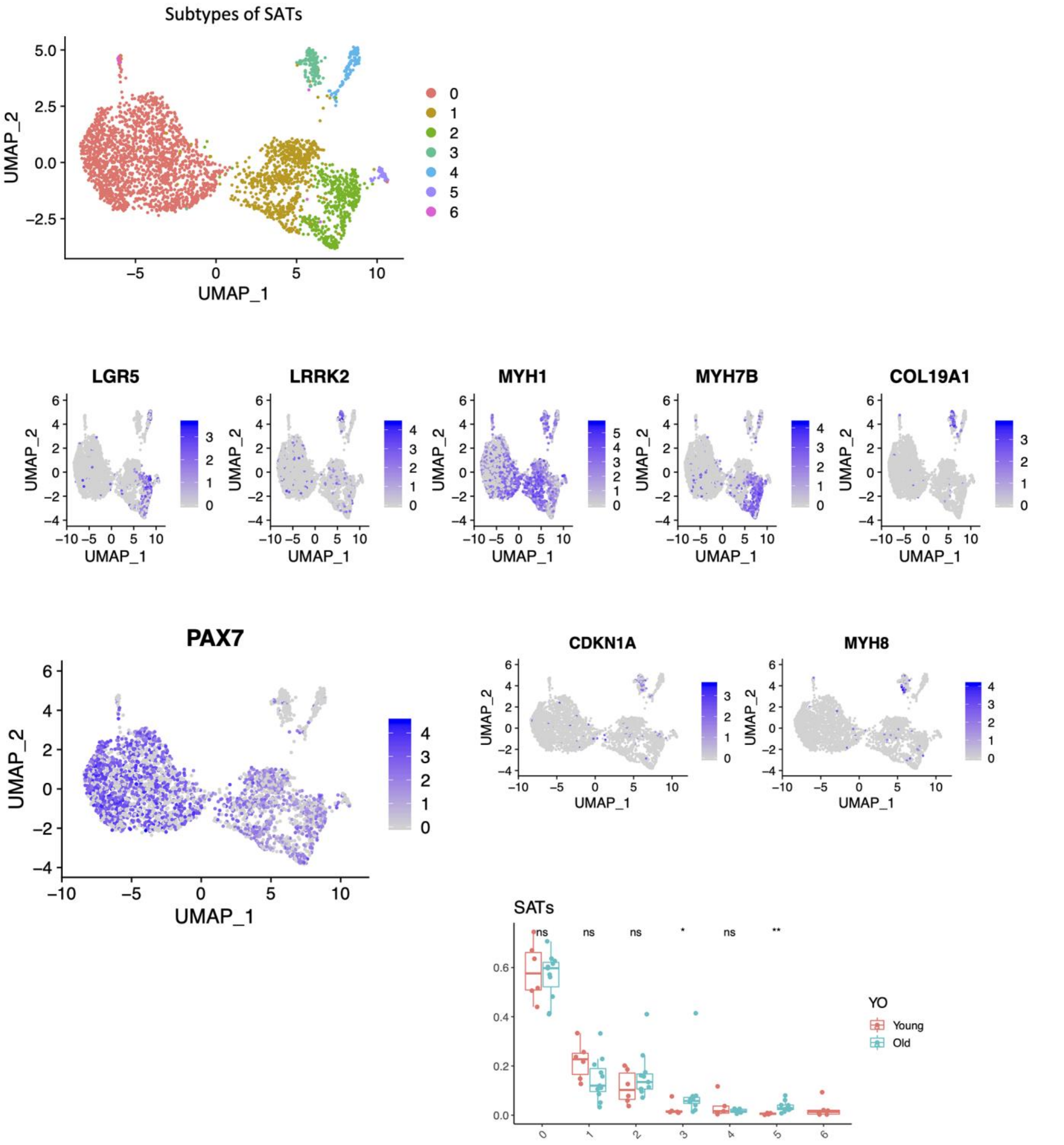

Supplementary Figure 6. Subtypes of SATs (UMAP, all samples). Markers expressed in different cell types. Difference in proportions between young and old for all subtypes. Significance of the *t*-test between young and old is shown at the top.

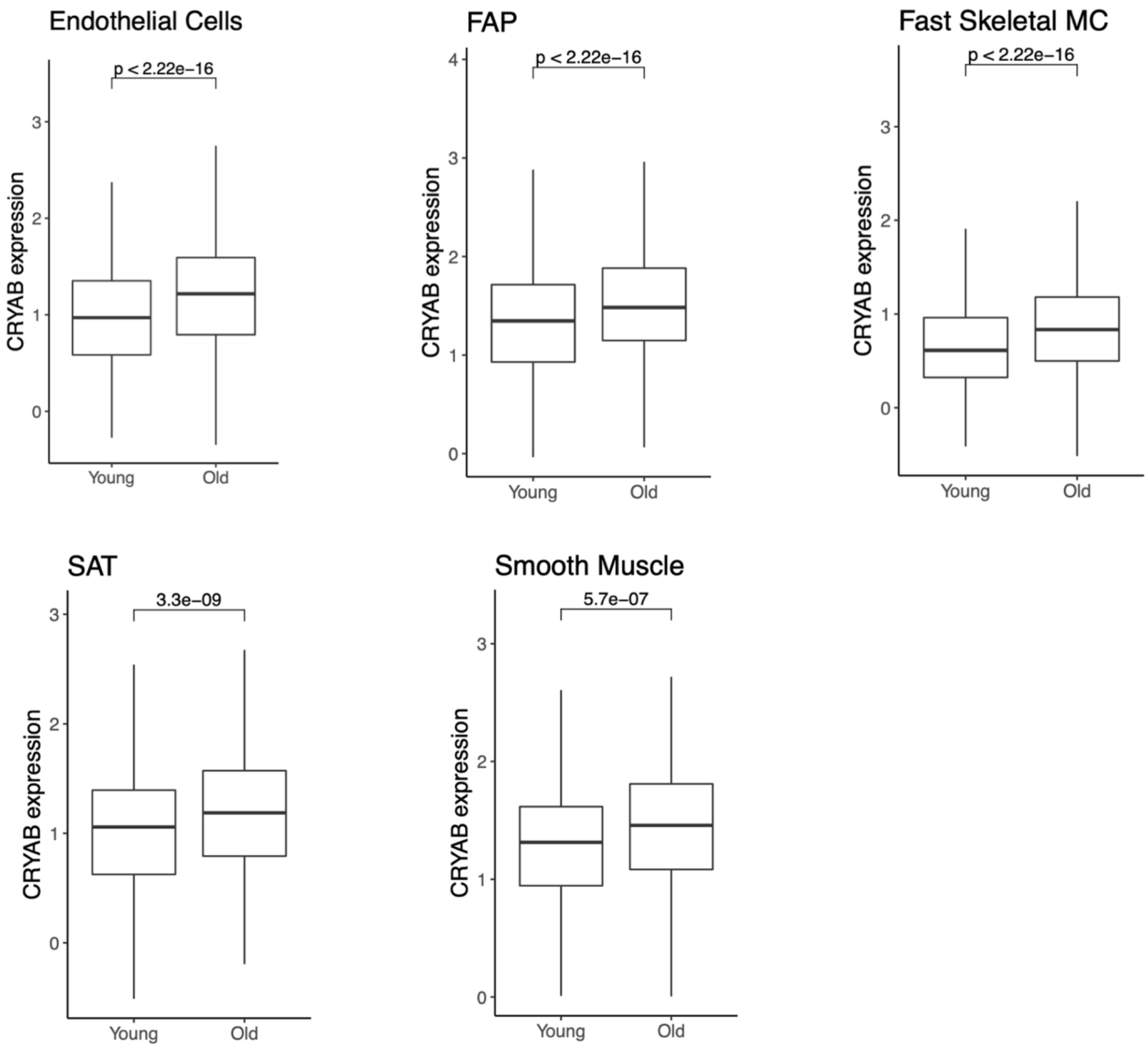

Supplementary Figure 7. CRYAB gene expression in single nuclei with age. A small but significant increase in CRYAB is seen within each cell type with age. Abbreviation: MC: muscle cells.
