# Supplementary material for: Single nuclei profiling identifies cell specific markers of skeletal muscle aging, frailty, and senescence
Source: Aging (Albany NY). 2022 Dec 13;14(23):9393–422. doi: 10.18632/aging.204435 (PMC9792217; doi:10.18632/aging.204435)
Supplement: Supplementary Tables 1 and 2 [file aging-14-204435-s003.pdf]

SUPPLEMENTARY TABLES

Supplementary Table 1. Mean (standard deviation) values for clinical parameters in the bulk cohort.

	Old healthy *N* = 29	Frail *N* = 24	Young *N* = 19
Age	72.4 (7.07)	76.9 (7.44)	22.1 (2.95)
6 min walk test (m/s)	1.10 (0.22)	0.97 (0.23)	1.19 (0.24)
Peak Torque (Nm)	196 (41.4)	145 (30.7)	290 (69.6)
Time Up and Go (sec)	7.56 (1.35)	8.80 (2.05)	6.08 (0.87)
SPPB Total	11.1 (1.03)	9.61 (1.95)	12.0 (0.00)
Grip Strengh	43.2 (8.54)	37.5 (8.85)	50.0 (9.89)
Biodex	142 (31.0)	112 (28.9)	176 (35.5)

Supplementary Table 2. Assessment of changes in bulk RNA-seq with clinical parameters.

Parameter	Up	Down
SPPB	RPL10P9, CGA, MAP7D2	
Grip Strengh	PPBP, SPAM1, SPATA17, LRRC65	
Time Up and Go	RPL10P9, GRP20, PPFIA3, IGFN1, GAS2L2	MTRNRL8, MTND4P24
6 min Walk Test	MTCYBP35, PP2R2B, CDK18, S100A2, CAMD5	
Biodex	GPR61, MAP7D2	COL19A1, MYCL, LMO2, MPZL2, PNPLA3, SLC47A2
Leg Press		PAX5, COL25A1, NPTX1, PNPLA3

Analysis of old vs. frail samples only. Clinical factors were binarized to good and bad performers if they were above or below the median. DEGs between good and bad performers are shown for 6min walk test, SPPB, ‘Time up and Go’, grip strength, Biodex and leg press with *P*-value < 0.01, abs (logFC) >2 are shown.
